# Beyond Haldane’s rule: Sex-biased hybrid dysfunction for all modes of sex determination

**DOI:** 10.7554/eLife.96652

**Published:** 2024-08-19

**Authors:** Asher D Cutter

**Affiliations:** 1 Department of Ecology & Evolutionary Biology, University of Toronto Toronto Canada; University of Lille Lille France; Max Planck Institute for Biology Tübingen Germany

**Keywords:** speciation, Haldane's rule, sex determination, hybridization, incompatibility, mating systems

## Abstract

Haldane’s rule occupies a special place in biology as one of the few ‘rules’ of speciation, with empirical support from hundreds of species. And yet, its classic purview is restricted taxonomically to the subset of organisms with heteromorphic sex chromosomes. I propose explicit acknowledgement of generalized hypotheses about Haldane’s rule that frame sex bias in hybrid dysfunction broadly and irrespective of the sexual system. The consensus view of classic Haldane’s rule holds that sex-biased hybrid dysfunction across taxa is a composite phenomenon that requires explanations from multiple causes. Testing of the multiple alternative hypotheses for Haldane’s rule is, in many cases, applicable to taxa with homomorphic sex chromosomes, environmental sex determination, haplodiploidy, and hermaphroditism. Integration of a variety of biological phenomena about hybrids across diverse sexual systems, beyond classic Haldane’s rule, will help to derive a more general understanding of the contributing forces and mechanisms that lead to predictable sex biases in evolutionary divergence and speciation.

## Introduction

**Figure 1.**
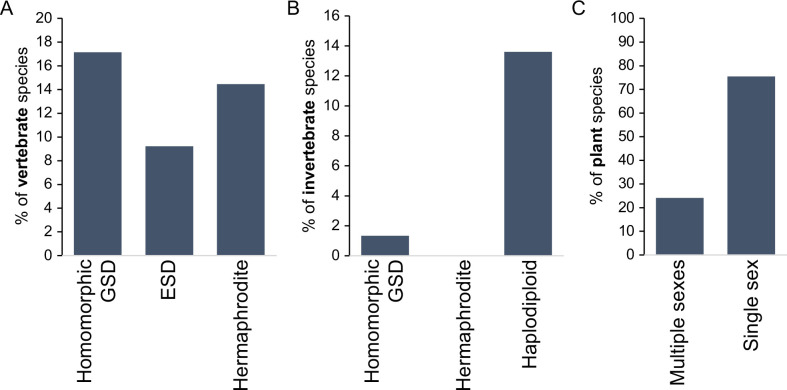
Incidence of different sexual modes in vertebrates (**A**), invertebrates (**B**), and plants (**C**). Vertebrate values out of 1475 species with information on the karyotype (homomorphic genetic sex determination, GSD) or 2145 species with information on sexual system Environmental sex determination (ESD), hermaphrodite. Invertebrate values out of 11914 species, with homomorphic value including any incidence of homomorphism and haplodiploidy excluding cases of paternal genome elimination; only 2 cases (0.02%) of hermaphroditism are indicated. Species with heteromorphic sex chromosomes are reported for 57% of species in both vertebrates and insects. Plant values out of 11038 species with information on the sexual system. Plant cases with single sex include hermaphrodite and monoecy (excludes apomixis); multiple sexes include dioecy, androdioecy, gynodioecy, andromonoecy, etc. Data was redrawn from Ashman et al., 2014; Bachtrog et al., 2014.

Haldane’s rule – disproportionate sterility, rarity, or inviability of the heterogametic sex in inter-species hybrids Haldane, 1922 – is touted, with good reason, as ‘one of the most general patterns in speciation biology (Schilthuizen et al., 2011).’ Hundreds of examples document excess hybrid male sterility (e.g. mammals and flies and nematodes) or excess hybrid female sterility (e.g. birds and butterflies), among diverse animal taxa and including plants (Laurie, 1997; Orr, 1997; Coyne and Orr, 2004; Schilthuizen et al., 2011; Watson and Demuth, 2012; Delph and Demuth, 2016; Fontdevila, 2016; Cutter, 2018). These patterns of post-zygotic reproductive isolation that are genetically intrinsic to organismal development in hybrids also often apply to sex-ratio biases among hybrids, typically due to sex-biased inviability. At least six evolutionary hypotheses receive support as compelling partial and non-exclusive explanations for the Haldane’s rule pattern (see below) (Laurie, 1997; Orr, 1997; Kulathinal and Singh, 2008; Presgraves, 2010a; Schilthuizen et al., 2011; Delph and Demuth, 2016; Cowell, 2023). And yet, many species of animals and plants with separate sexes lack heteromorphic sex chromosomes (Figure 1), and many others are comprised of hermaphrodites (Bachtrog et al., 2014), rendering Haldane’s rule irrelevant in a simple application to such taxa.

**Figure 2.**
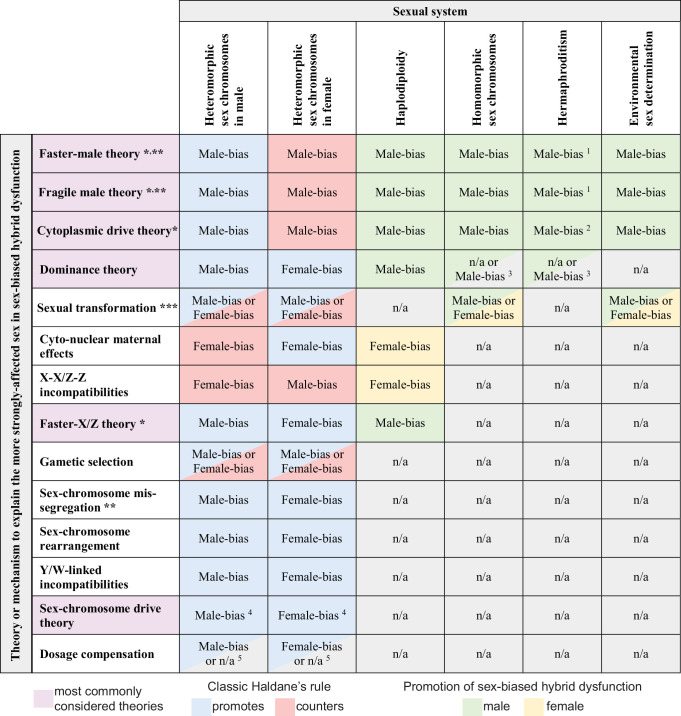
Predicted contributions to sex-biased hybrid dysfunction in different sexual systems for alternative hypotheses that aim to explain the classic Haldane’s rule pattern. Predictions for haplodiploidy applies to comparisons involving F1 females with males from F2 and later-generation hybrids (Laurie, 1997; Koevoets and Beukeboom, 2009; Bendall et al., 2023). * mechanisms sometimes subsumed under the umbrella of ‘faster heterogametic sex theory’ (Kulathinal and Singh, 2008); ** primarily or only expected to affect hybrid sterility; *** details of sex determination pathway disruption may predispose taxa of a given sexual system to a particular direction of sex bias in absence or rarity; ^1^ hybrid dysfunction biased toward male gametes (sperm, pollen) and accessory structures; ^2^ cytoplasmic male sterility in F1 hybrids may not serve as a reproductive isolating barrier (Rieseberg and Blackman, 2010); ^3^ applies to haploid gametophytic phase (e.g. following pollen germination) for taxa like plants with active haploid stages of male gametes; ^4^ does not apply in XO or ZO systems (Coyne et al., 1991); ^5^ does not apply to systems lacking global dosage compensation mediated by downregulation of both sex chromosome copies in the homogametic sex.

Some hypotheses to explain Haldane’s rule, however, may be extended to or tested with those taxa that do not have heteromorphic sex chromosomes (Figure 2; Presgraves and Orr, 1998; Koevoets and Beukeboom, 2009; Schilthuizen et al., 2011). Diverse taxa contain distinct sexes with individuals predisposed to developing small gametes (male sperm) or large gametes (female eggs), or a sex capable of producing both types of gamete (hermaphrodites), through an array of mechanisms that do not involve heteromorphic sex chromosomes (Bell, 1982; Bachtrog et al., 2014). In interspecies hybrids between taxa lacking heteromorphic sex chromosomes, the sexes do indeed often differ in the extent to which they display sterility, inviability, or other dysfunctional phenotypes (Presgraves and Orr, 1998; Malone and Fontenot, 2008a). Moreover, male versus female sexual structures of hermaphrodites often show different susceptibility to developmental disruption in inter-species hybrids (Rieseberg and Blackman, 2010; Baack et al., 2015; Fishman and Sweigart, 2018; Cutter, 2019).

I propose that Haldane’s rule, in the strict sense of taxa with heteromorphic sex chromosomes, be considered a special case of the more general phenomenon of sex-biased hybrid dysfunction (Box 1). By testing alternative hypotheses for sex-biased hybrid dysfunction that may also apply to taxa with homomorphic sex chromosomes, environmental sex determination, haplodiploidy, or hermaphroditism, we may derive a more comprehensive understanding of the forces and mechanisms that lead to predictable sex biases in evolutionary divergence and speciation.

Box 1. **Classic Haldane’s rule and a broadening of perspective.**

J.B.S. Haldane defined in 1922 the oft-quoted pattern known as Haldane’s rule: ‘When in the F1 offspring of a cross between two animal species or races one sex is absent, rare, or sterile, that sex is always the heterozygous sex’ (p. 108). This principle is taken to apply to crosses between any kind of sexually-reproducing taxa, not just animals (Brothers and Delph, 2010; Kasjaniuk et al., 2019), and ‘heterozygous’ in this context is understood to indicate heteromorphic sex chromosomes in organisms with genetic sex determination (also termed ‘heterogametic’). I consider this scenario to encapsulate ‘classic Haldane’s rule’ in the strict sense, and as it is generally conceived and cited in the speciation literature.

A number of authors have pointed out that sex biases in interspecies hybrid dysfunction appear in circumstances that do not conform neatly to the classic Haldane’s rule situation. For example, Presgraves and Orr, 1998 deduced that faster male evolution likely contributes to the pattern of male-biased hybrid sterility in *Aedes* mosquitoes that have homomorphic sex chromosomes. These authors later pointed out that male structures in hermaphrodite or monoecious plants (i.e. pollen or pollen-bearing structures) might also suffer disproportionate dysfunction in hybrids (Orr and Presgraves, 2000). Indeed, for example, hybrid male (pollen or sperm) sterility is especially strong in hermaphroditic *Mimulus* monkeyflowers (Fishman and Willis, 2001) and *Argopecten* scallops (Yu et al., 2023). Subsequently, Koevoets and Beukeboom, 2009 described how a modified version of Haldane’s rule is pertinent to haplodiploids. Analysis of hybrids beyond the F1 generation also informs sex-biases in important ways (Demuth et al., 2014). These various issues have been characterized as ‘Haldane’s rule-like’ patterns (Schilthuizen et al., 2011).

For consistency, I refer to all of the circumstances of sex biases in hybrid dysfunction as pertinent to a generalized view of Haldane’s rule, regardless of the sexual system (Figure 2). Despite this broader perspective on sex-biased hybrid dysfunction, it nonetheless depends on sexual reproduction and so excludes obligate asexual species. The sex bias may plausibly influence hybrid fertility or hybrid viability, among other traits, depending on the sexual system and mechanism under consideration. Consequently, a broad view of Haldane’s rule poses the hypothesis that, ‘In the F1 or later generation hybrids that follow from a cross between two populations with partial reproductive isolation, dysfunctional development differs consistently between the sexes or sexual functions’.

## Theories to explain sex-biased hybrid dysfunction

A wide variety of theories have emerged to explain Haldane’s rule (Schilthuizen et al., 2011; Delph and Demuth, 2016; Cowell, 2023), some of which extend beyond taxa with heteromorphic sex chromosomes. The emergent consensus holds that each of these models is important to different degrees in different taxa to make Haldane’s rule a composite phenomenon (Orr, 1993b; Wu and Davis, 1993; Schilthuizen et al., 2011; Cowell, 2023). Thus, there is no single cause of Haldane’s rule even in the strict sense, further encouraging a generalized view of causes responsible for sex bias in hybrid dysfunction. Here, I first summarize the rationales for six common explanations for Haldane’s rule. I also introduce several additional rarer explanations, and introduce their implications for alternative sexual systems, before considering alternative sexual systems directly in a subsequent section.

### Dominance theory

Dominance theory, proposed by Muller, 1940, provides one of the simplest potential explanations for classic Haldane’s rule. If loci that induce Dobzhansky-Muller incompatibilities (DMIs) tend to act recessively in hybrids (Box 2), as appears to be the case at least in *Drosophila* (True et al., 1996; Presgraves, 2003; Masly and Presgraves, 2007), then their linkage to sex chromosomes would unmask their negative effects of epistasis in the hemizygous sex to yield hybrid dysfunction (Orr, 1993a; Turelli and Orr, 1995; Orr and Turelli, 1996). Consequently, males in taxa with heteromorphic XY and XO chromosomal sex determination systems would suffer a more pronounced incidence of hybrid sterility and inviability (females in ZW and ZO systems). This pattern of sex bias is expected to be especially robust when sex chromosomes comprise a greater fraction of the genome and when DMIs involve more loci (Orr and Turelli, 1996).

Box 2. **Dobzhansky-Muller incompatibilities and explanations for Haldane’s rule.**

The Bateson-Dobzhansky-Muller model invokes multi-locus epistasis with negative fitness effects to explain how diverging populations can evolve intrinsic reproductive isolation as an incidental byproduct of genetic drift or adaptive evolution (or genetic drive) (Orr, 1995; Orr, 1996). The idea is that novel-derived alleles arise and evolve at each locus in each descendant population independently, and so there is no loss of fitness within any given population as the alleles arise and become fixed. When the novel alleles are given the opportunity to interact for the first time, in a hybrid organism, they may fail to interact properly. Such incompatible genetic interactions in hybrids, termed Dobzhansky-Muller incompatibilities (DMIs), can cause dysfunctional development that manifests as hybrid sterility or hybrid inviability. While DMIs often are depicted in their simplest form involving two loci (Box 2—figure 1), they may commonly involve ‘complex epistasis’ of more than two loci (Presgraves, 2010a; Phadnis, 2011; Satokangas et al., 2020). Should the derived alleles act recessively in hybrids, then their effects will be apparent in F1 organisms only when linked to hemizygous chromosomes in hybrid genomes that contain DNA sourced from both parents, such as involving sex chromosome linkage. Dominance theory proposes that such DMIs will lead to sex-biased hybrid dysfunction due to sex-chromosome linkage (classic Haldane’s rule), provided that incompatibility loci most often act recessively in hybrids (Orr, 1993a; Turelli and Orr, 1995; Orr and Turelli, 1996). Most other explanations for Haldane’s rule also appeal to genic DMIs as providing the genetic source of hybrid dysfunction, with the idea of sex-chromosome mis-segregation as one notable exception.

**Box 2—figure 1.**
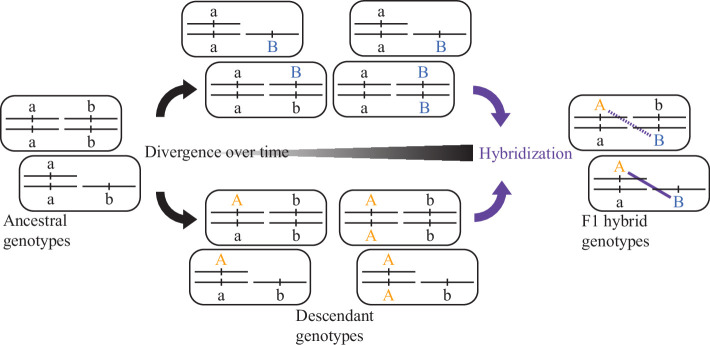
Evolution of a two-locus DMI. Independent accumulation of derived alleles (autosomal orange A, sex-chromosomal blue B) in different populations for distinct loci can lead to incompatible genetic interactions between them upon hybridization (purple). When one of the loci is linked to a sex-chromosome, then recessive-acting incompatibility loci can reveal their fitness effects in the F1 generation of hybrid individuals hemizygous for that sex chromosome.

The logic of dominance theory also applies to haplodiploid systems in which haploid males develop from unfertilized eggs, with some additional nuance due to the absence of X-autosome DMIs (Koevoets and Beukeboom, 2009; Schilthuizen et al., 2011). But dominance theory does not predict sex-biased hybrid dysfunction in taxa with homomorphic sex chromosomes, environmental sex determination, or hermaphroditism (excepting sex-biases in the active haploid sexual phases of the life cycle, e.g. plant gametophytes) (Figure 2). Despite the prominent role of dominance theory in Haldane’s rule among placental mammals, it is insufficient as an explanation for sex differences in marsupials due to the mechanism of dosage compensation that silences the paternal copy of the X-chromosome in females (Watson and Demuth, 2012). Moreover, if some taxa are predisposed to partially-dominant rather than recessive mutations (as they act within hybrid individuals), on average, perhaps due to peculiarities of genetic architecture, then dominance theory in such taxa would be expected to work in opposition to classic Haldane’s rule (Turelli and Orr, 1995). For example, the relative incidence of adaptive evolution from new mutations versus standing variation will affect the realized distribution of dominance among fixed substitutions (‘Haldane’s sieve’) (Orr and Betancourt, 2001), as will migration during local adaptation (Zwaenepoel et al., 2024) or the incidence of selection on haploid expression during the gametophytic phase of life cycles (Peters and Weis, 2018). Notably, however, the dominance relationships of alleles during the within-species population genetic processes resulting in substitutions need not be equivalent to dominance relationships expressed from hybrid genomes. Despite the inescapable conclusion for most heterogametic taxa that ‘dominance plays a more universal and thus fundamental role in Haldane’s rule’ than do other explanations (Orr, 1997), dominance is largely irrelevant to explaining sex-biased hybrid dysfunction in non-heterogametic and some other taxa.

### Faster X/Z theory

Theory predicts that genes linked to the X or Z sex chromosomes will experience more rapid molecular evolution under some population genetic conditions (Charlesworth et al., 1987; Charlesworth et al., 2018). Namely, ‘faster X/Z’ evolution can occur if adaptation results from new mutational input with recessive effects, especially when they are beneficial to male fitness, or in combination with male-biased mutation rates or greater variance in male versus female reproductive success (Orr, 1993a; Turelli and Orr, 1995; Presgraves and Meiklejohn, 2021). Provided that each mutational substitution confers some probability of contributing to a DMI in hybrids (Orr and Turelli, 2001), such faster X/Z evolution could lead to disproportionate involvement of these sex chromosomes in hybrid dysfunction i.e., a large X effect (Orr, 1989b; Coyne, 1992) that exaggerates the Haldane’s rule pattern expected from dominance theory. In addition, should genes with male-biased expression and faster evolution from new mutational input more often be linked to the sex chromosomes, then the Haldane’s rule pattern could emerge as a byproduct (Orr, 1997). The molecular evolution of X/Z-linked loci shows mixed, if often supporting, evidence across taxa for accelerated divergence (Kulathinal and Singh, 2008; Meisel and Connallon, 2013; Delph and Demuth, 2016). In addition to accelerated coding sequence evolution, gene expression divergence that likely reflects non-coding regulatory sequence evolution also appears to accumulate more rapidly for X-linked loci in mammals and *Drosophila* (Meisel and Connallon, 2013; Coolon et al., 2015). The faster X/Z theory, of course, makes no prediction about sex-biased hybrid dysfunction in taxa with an indistinguishable karyotypic make-up between the sexes (Figure 2).

### Sex-chromosome drive theory

Meiotic drive elements linked to a sex chromosome (X or Y, Z or W) can invade populations and skew sex ratios through a variety of mechanisms, which imposes strong selection for alleles at other loci capable of acting as suppressors of drive (Hurst and Werren, 2001; Jaenike, 2001; Werren, 2011; Rice, 2013; Lindholm et al., 2016). This situation of arms race coevolution can lead to a special kind of faster X/Z evolution and the formation of DMIs that generate classic Haldane’s rule in interspecies hybrids, with the DMIs arising as a result of hybrids inheriting sex-linked drive alleles and mismatched suppressor alleles at other loci (Frank, 1991; Hurst and Pomiankowski, 1991). Meiotic drive systems frequently influence meiosis in males (or gamete maturation; see below discussion of gametic selection cf. ‘true drive’) (Jaenike, 2001; McDermott and Noor, 2010), so incompatibility from the ‘mutual imbalance of meiotic drive genes’ is predisposed to disrupt fertility in hybrid males disproportionately (Hurst and Pomiankowski, 1991). Driving elements are implicated in hybrid male sterility in several combinations of *Drosophila* species and in stalk-eyed flies (McDermott and Noor, 2010; Phadnis, 2011; Patten, 2018). Thus, sex-chromosome drive theory interacts with male-centric theories (faster male, fragile male) as well as other sex chromosome-centric theories (faster X/Z). Incompatibilities arising from co-evolution from the sex-chromosome drive, however, might be expected to disproportionately impact the heterogametic sex regardless of whether they are male (Tao and Hartl, 2003). To the extent that drive-mediated coevolution tends to generate X-Y (or Z-W) incompatibilities, there is some speculation that sex-chromosome drive might be less likely to underlie sex-biased hybrid dysfunction in taxa with XO sex determination, in addition to taxa that lack sex chromosomes altogether (Johnson, 2010; Moran et al., 2017). Sex-chromosome drive as a source of sex-biased hybrid dysfunction, of course, does not extend to taxa lacking sex chromosomes.

Transposable elements (TE) also present a form of genetic drive with the potential to generate DMIs in hybrids due to mismatch with suppressors of their activity (Hurst and Pomiankowski, 1991; Castillo and Moyle, 2012; Ågren, 2013; Cutter, 2023b), and may accumulate disproportionately on sex chromosomes (Peona et al., 2021). As a consequence, TE-mediated sex biases in hybrid dysfunction could arise if TE activity, or mismatches in TE suppression, involves sex-chromosome linkage (see faster-X/Z theory) or interferes with sex-dependent developmental genetic architecture (see fragile male theory below).

Selfish genetic elements that can invade populations by incapacitating those post-meiotic gamete cells that lack the element, sometimes referred to as ‘gamete-killer’ meiotic drivers, differ from mechanisms of ‘true drive’ that influence meiosis to bias the transmission process itself (McDermott and Noor, 2010; Patten, 2018; Cutter, 2023b). Such cases of gamete-killers are a form of gametic selection, which can be challenging to distinguish from ‘true drive’ (Scott et al., 2018). Gametic selection exacerbates Haldane’s rule in terms of a hybrid male rarity in some *Caenorhabditis* nematodes due to a competitive advantage in fertilization by X-bearing sperm over nullo-X sperm (Bundus et al., 2015). In principle, gametic selection also could lead to an advantage of Y-bearing sperm. Gametic selection, irrespective of selfish genetic elements, is especially prominent in plants in pollen competition (Lankinen and Karlsson Green, 2015; Immler, 2019). Similarly, gametophytic haploid selection presents itself as an especially important factor in those organisms with extended gametophytic stages of the life cycle, as in some bryophytes and algae (Haig, 2016; Beaudry et al., 2020; Charlesworth, 2022).

### Cytoplasmic drive theory

Divergence of cytoplasmic drive elements between species also can lead to Haldane’s rule (Hurst and Pomiankowski, 1991). Again, sex-limited inheritance of cytoplasmic drive elements that are linked to plastid genomes or vertically-inherited endosymbiotic bacterial genomes skew sex ratios and select for suppressor alleles, setting up arms-race coevolution (Werren, 2011). Mismatched driver and suppressor genotypes in hybrids can manifest as hybrid sterility or inviability, as documented in *Drosophila* and other insects (Hurst and Pomiankowski, 1991; McDermott and Noor, 2010; Miller et al., 2010; Shropshire et al., 2020), in some cases also contributing to reciprocal cross asymmetry (Darwin’s corollary to Haldane’s rule) (Jaenike et al., 2006; Turelli and Moyle, 2007). Similarly, the cytoplasmic endosymbiont *Wolbachia* can lead to reproductive isolation with sex-biased effects, with evidence from *Tribolium* beetles, mushroom-feeding *Drosophila*, and haplodiploid *Nasonia* wasps (Wade et al., 1995; Shoemaker et al., 1999; Bordenstein and Werren, 2007).

Hermaphroditic plants are well-known to experience cytoplasmic hybrid incompatibility as a source of sterility for hybrid pollen, often arising from mitochondrial rearrangements (Frank, 1989; Rieseberg and Blackman, 2010). At face value, then, one can interpret such cases as a source of Haldane’s rule when examining F1 hybrids. However, the genetic drive that promotes the evolution of such cytoplasmic male sterility (CMS) alleles in the first place may subvert their potential to provide long-term reproductive barriers pertinent to speciation by promoting outcrossing and gene flow (Rieseberg and Blackman, 2010; Ågren, 2013; Sweigart et al., 2019).

These cases of haplodiploid and hermaphrodite sexual systems highlight how drive as a factor can contribute to sex-biased hybrid dysfunction in taxa lacking heteromorphic sex chromosomes. The fact that the magnitude of cytoplasmic-mediated dysfunction caused by *Wolbachia* is temperature-sensitive (Shropshire et al., 2020) suggests the speculative possibility that systems with environmental sex determination also might exhibit predictable consequences of cytoplasmic incompatibility in hybrids.

### Faster male theory

Models of sexual selection and sexually antagonistic selection often predict the more rapid evolution of male reproductive traits and the genes that underlie them (Grath and Parsch, 2016; Kasimatis et al., 2017; Swanson and Vacquier, 2002). This logic extends to hybrid dysfunction by again presuming that each new mutational substitution increases the likelihood of contributing to a DMI. Consequently, faster male theory predicts that hybrid males will suffer disproportionate hybrid dysfunction irrespective of the mode of sex determination (Wu and Davis, 1993). Because genes controlling the development of the gonad and other reproductive structures provide prime sources of sex-biased gene expression and targets of sexual selection, the faster male hypothesis is thought to more readily explain sex-biased sterility than the inviability of hybrids (Wu and Davis, 1993; Wu et al., 1996). Even hermaphrodite systems can experience sexual selection and sexual conflict to promote the evolution of male gametes and reproductive structures (Abbott, 2011; Lankinen and Karlsson Green, 2015; Schärer et al., 2014; Cutter, 2019). Consequently, this theory applies to sex-biased hybrid dysfunction irrespective of the mechanism of sex determination and makes no assumptions about the dominance of incompatibility alleles (Figure 2). In addition, genes with sex-biased expression will experience weaker purifying selection, and so also may evolve faster than other loci due to mutation accumulation by genetic drift (Demuth and Wade, 2007b; Dapper and Wade, 2016; Dapper and Wade, 2020). Molecular evolutionary evidence generally supports the more rapid divergence of coding sequence and of expression profiles for genes with male-biased expression (Swanson and Vacquier, 2002; Ellegren and Parsch, 2007; Grath and Parsch, 2016). Regardless of whether faster male evolution might result from directional selection or relaxed selection, however, it will act in opposition to other forces that promote the classic Haldane’s rule pattern in those taxa with female heterogamety (Turelli and Orr, 1995; Figure 2). If sufficiently strong, then faster male evolution could attenuate or reverse the magnitude of Haldane’s rule in some female-heterogametic organisms to help explain exceptions to classic Haldane’s rule, as in *Xenopus* frogs (Malone and Michalak, 2008b; Table 1).

**Table 1.** Exemplar taxa and exceptional examples with experimental evidence that is informative about sex biases in hybrid dysfunction for different sexual systems.

Sexual system	Exemplar taxa *	Exceptional examples **	Evidence informative to sex biases in hybrid dysfunction ***	Review references
Heteromorphic sex chromosomes in male	Many insects (e.g. *Drosophila*, Lepidoptera), mammals, nematodes; *Silene* plants	*Teleogryllus* (field crickets; XX/XO)	Pervasive male-biased hybrid sterility & inviability; support for multiple mechanisms affecting Haldane’s rule	Delph and Demuth, 2016; Fontdevila, 2016; Moran et al., 2017
Heteromorphic sex chromosomes in female	Many insects, birds, nematodes	*Xenopus* (clawed frogs; ZW/ZZ)	Pervasive female-biased hybrid sterility & inviability; support for multiple mechanisms affecting Haldane’s rule	Malone and Michalak, 2008b; Delph and Demuth, 2016; Fontdevila, 2016
Haplodiploidy	*Nasonia* (wasps), *Formica* (ants), *Tetramorium* (ants), *Tetranychus* (mites)	*Neodiprion* (sawflies)	Sex-biased hybrid inviability more common than hybrid sterility; support for cytoplasmic drive	Koevoets and Beukeboom, 2009; Bendall et al., 2023
Homomorphic sex chromosomes	*Aedes* (mosquitoes), *Tigriopus* (copepods), many amphibians, and fish	*Bufo* (toads)	Heterogeneous support for sex-biases across taxa; higher female inviability and higher male sterility in *Bufo*; support for faster male and/or fragile male theories (*Aedes*, *Bufo*)	Presgraves and Orr, 1998; Malone and Fontenot, 2008a; Lima, 2014; Dufresnes and Crochet, 2022
Hermaphroditism	Many plants (e.g. *Mimulus*, *Solanum*, *Helianthus*, *Arabidopsis*), molluscs, nematodes	*Argopecten* (scallops)	Pervasive sterility of male function (pollen, sperm); support for cytonuclear incompatibilities	Rieseberg and Blackman, 2010; Yu et al., 2023
Environmental sex determination	Many turtles and fish, some lizards	*Lepomis* (sunfish)	Male-biased sex ratios	Bolnick, 2009

* taxonomic scale varies across study systems used in speciation research; ** recent or rare well-developed example system; *** the commonness or rarity of sex-biased hybrid dysfunction across taxa remains to be determined for most sexual systems that lack heteromorphic sex chromosomes.

### Fragile male theory

The initial proposition of faster male theory for Haldane’s rule also posited that spermatogenesis might be especially prone to disruption to also make male hybrids especially vulnerable to hybrid dysfunction (Wu and Davis, 1993). Here, I separate this hypothesis regarding the fragility of male developmental mechanisms as a conceptually distinct idea relative to faster male theory in the strict sense of rapid divergence of genes and traits with male-biased expression that result from sexual selection or conflict-driven rapid evolution (Laurie, 1997; Cutter and Bundus, 2020).

Although no explicit mechanism was proposed to explain the fragility of male development (Wu and Davis, 1993), disruption of meiotic checkpoints are more prone to induce sterility in spermatogenesis than oogenesis in some organisms (Hunt and Hassold, 2002; Malone and Michalak, 2008c). The sexes also experience intrinsic sex differences in gene regulatory network architecture, at least in some contexts, that could potentially underlie different sensitivities to perturbation. For example, spermatogenesis developmental programs depend primarily on transcriptional regulation in *C. elegans* nematodes, whereas post-transcriptional regulation dominates for other germline genes (Merritt et al., 2008). It may be the case that post-transcriptional regulation confers greater robustness to genetic networks (McManus et al., 2014). In addition, the architecture in terms of genetic network size, connectivity, or modularity that underlies the development of male traits might predispose them to low robustness in the face of genetic or environmental perturbations as experienced in hybrid organisms (Cutter and Bundus, 2020; Cutter, 2023a). For example, genes with sex-biased expression in nematodes also differ in how regulatory divergence has accumulated changes to *cis*-acting versus *trans*-acting regulatory factors that could predispose one sex to dysfunctional control of tissue development in hybrids (Sánchez-Ramírez et al., 2021). Thus, even if genes with male-biased expression do not evolve any faster than other genes, genetic network architecture may lead to disproportionately perturbed male fertility or viability in hybrids.

While faster male and fragile male hypotheses are typically applied to hybrid sterility (Wu and Davis, 1993; Wu et al., 1996), sex differences in developmental genetic mechanisms might extend these explanations to hybrid inviability. For example, extreme temperature conditions exacerbate the magnitude of Haldane’s rule for hybrid male inviability in both insects and nematodes (Wade et al., 1999; Bundus et al., 2015). If these properties are general to male gametogenesis and the development of related traits, then the fragile male hypothesis will apply to sex-biased hybrid dysfunction in taxa irrespective of sexual system (Figure 2).

### Other hypotheses to explain sex biases in hybrids

Despite the prominence of the aforementioned hypotheses to explain Haldane’s rule, several other ideas (Figure 2) have been proposed over the years with varying degrees of evidentiary support (Cowell, 2023). It is valuable to consider their potential influence in any given system, given that no single explanation accounts for all observations of sex-biased hybrid dysfunction (Coyne, 2018). For example, disruptions to sex determination developmental pathways can contribute to Haldane’s rule by **sexual transformation** (Sturtevant, 1920; Haldane, 1922), as observed in hybrids of some nematode species in which XO hybrid individuals develop as females rather than as males (Baird, 2002). Sexual transformation can help explain the absence or rarity of a sex, but does not easily account for sex-biased sterility. Moreover, the expected directionality of sex bias caused by perturbed genetic pathways governing sex determination will depend on the details of genetic pathway structure for the taxa under consideration. Notably, cytoplasmic drive elements also sometimes operate through sexual transformation (Legrand et al., 1987).

Perhaps related to Haldane’s rule by sexual transformation in some circumstances, disrupted mechanisms of **dosage compensation** and meiotic silencing of unsynapsed chromatin can contribute to Haldane’s rule in some species (Orr, 1989a; Johnson and Lachance, 2012). These circumstances include situations in which neo-sex chromosomes are evolving greater heteromorphism (Filatov, 2018). However, disruption to some mechanisms of dosage compensation could oppose classic Haldane’s rule (Laurie, 1997). Moreover, distinct mechanisms of dosage compensation also are important for understanding other models, such as dominance theory and the fragile male hypothesis (Johnson and Lachance, 2012). For example, the details of dosage compensation preclude dominance as a cause of Haldane’s rule in marsupials because they show paternal X-chromosome silencing in females rather than the mosaic silencing seen in placental mammals (Watson and Demuth, 2012). The putative lack of global dosage compensation in taxa like the Lepidoptera also rules out a contribution of dosage compensation as an explanation in some organisms (Presgraves, 2002).

Gene movement from sex-chromosomes to autosomes as a source of divergence between species, even in the absence of any change in gene function, can lead to Haldane’s rule (Haldane, 1932; Zeng, 1996; Moyle et al., 2010). The impact of such **sex-chromosome rearrangement**, translocation, and duplication may also be compounded by greater densities of sex-biased genes on the sex chromosomes, mediating hybrid dysfunction due to null genotype effects, dosage effects, and X/Z-inactivation effects (Moyle et al., 2010). Consequently, any effects of sex-chromosome rearrangements on viability and fertility in F1 may depend on the degree of post-meiotic expression by gametes, the details of dosage compensation mechanisms, or the sensitivity of sex-chromosome inactivation pathways. Sex chromosome rearrangements are implicated in Haldane’s rule in *Rumex* plants, with impacts on male (pollen) fertility in particular (Kasjaniuk et al., 2019).

Because any DMI interactions that involve loci linked to Y or W chromosomes would necessarily be sex-specific, such interactions can provide an alternative explanation for classic Haldane’s rule that is independent of dominance theory (Turelli and Orr, 1995; Delph and Demuth, 2016). Despite evidence for such **Y/W-linked incompatibilities** in some systems (Turelli and Orr, 1995; Cocquet et al., 2012; Cowell, 2023), this idea cannot explain sex-biased hybrid dysfunction in XO/ZO systems or in taxa lacking heteromorphic sex chromosomes. DMIs involving Y/W-linked genes are expected to arise more readily in taxa with less-degenerated Y/W-chromosomes (Presgraves, 2002). DMIs resulting from **X-X or Z-Z interactions**, however, will tend to oppose Haldane’s rule in the F1 (Laurie, 1997).

While most explanations for Haldane’s rule invoke DMIs, **sex-chromosome mis-segregation** in F1s provides another possible contributor that does not depend on genic interactions per se (Haldane, 1922; Forsdyke, 2000; Demuth et al., 2014). Perhaps faster-X/Z evolution, elevated translocations off of sex chromosomes (Moyle et al., 2010), or centromeric divergence could lead to special susceptibility of sex chromosomes to errors in pairing or segregation in the heterogametic sex. It is possible that both genic DMIs and chromosomal segregation defects could act in concert to contribute to Haldane’s rule, especially in taxa capable of forming hybrids in spite of extensive DNA sequence divergence. There is some evidence consistent with sex-chromosome segregation defects in plant hybrids (Demuth et al., 2014), though this idea to explain Haldane’s rule does not extend beyond taxa with heteromorphic sex chromosomes.

Transgenerational parental effects also can influence the manifestation of Haldane’s rule through their interaction with sex chromosomes (Sawamura, 1996), acting distinctively from cyto-nuclear incompatibilities due to drive elements or endosymbionts (Turelli and Orr, 2000). **Maternal effects** in male-heterogametic systems, in particular, can act counter to classic Haldane’s rule to help explain exceptions to the expected pattern, whereas maternal effects contribute to Haldane’s rule in female-heterogametic taxa from any cyto-Z incompatibilities (Turelli and Orr, 2000). Paternal effects presumably would act in a reciprocal way, though I know of no explicit consideration of paternal effects on Haldane’s rule. DMIs that arise from plastid-encoded loci as plastid-Z incompatibilities in female-heterogametic taxa, however, also could contribute to hybrid female dysfunction as an additional source of dominance effects for the hemizygous Z-chromosome in hybrid females, beyond standard Z-autosome incompatibilities (Presgraves, 2002).

### Integrating sex-biased hybrid dysfunction across sexual systems

For many years, researchers have acknowledged the idea that explanations for Haldane’s rule may also explain patterns of reproductive isolation beyond just those taxa with heteromorphic sex chromosomes (Presgraves and Orr, 1998; Orr and Presgraves, 2000; Koevoets and Beukeboom, 2009; Schilthuizen et al., 2011). Here I will consider how different proposed explanations for classic Haldane’s rule intersect with the sexual systems of haplodiploidy, genetic sex determination (GSD) with homomorphic sex chromosomes, environmental sex determination (ESD), and hermaphroditism.

### Haplodiploidy (haploid arrhenotoky)

Sex differences in ploidy are found in approximately 15% of arthropod species (Normark, 2003; Koevoets et al., 2012b; de la Filia et al., 2015), and also are observed in some rotifers and nematodes (Mable and Otto, 1998). Haldane recognized such organisms as displaying ‘extreme cases of the normal type’ (p. 101) (Haldane, 1922) and relevant to the Haldane’s rule pattern. In terms of transmission genetics, the entire genome is analogous to an X-chromosome (or set of X-chromosomes) in species with XO sex determination (or, e.g. X_1_X_2_OO), a genome that lacks autosomes entirely.

Consequently, following an interspecies cross, hybrid males will contain genetic material from both species only in F2 and later generations as a result of meiotic recombination and segregation in F1 females (Koevoets and Beukeboom, 2009; Schilthuizen et al., 2011; Bendall et al., 2023). This feature requires that tests for sex biases in hybrid dysfunction must consider multiple cross-generations, which also means that sex bias cannot be assessed in haplodiploid systems for which F1 hybrid females are sterile or inviable. Sex-biased hybrid dysfunction in haplodiploids is expected only for recessive-acting DMIs (Koevoets and Beukeboom, 2009). Because the entire genome is effectively sex-linked in haplodiploids, it has been proposed that the faster-X/Z theory predicts stronger postzygotic isolation and reinforcement in haplodiploid than in diplodiploid taxa (Koevoets and Beukeboom, 2009), with support from evolutionary simulations (Bendall et al., 2022). Faster-X/Z theory predicts sex-biased effects in hybrids only when coupled to the more rapid evolution of genes with male-biased expression (Koevoets and Beukeboom, 2009), making it difficult to discriminate between faster-male and faster-X/Z theory in haplodiploids.

The lack of autosomes means that some explanations for classic Haldane’s rule should not apply to haplodiploids (e.g. sex-chromosome rearrangement, dosage compensation), and that some predictions require modification (Koevoets and Beukeboom, 2009; Bendall et al., 2023). For example, because all DMIs in haplodiploids effectively involve X-X interactions that ought to oppose classic Haldane’s rule (Laurie, 1997), we might expect such taxa to often provide examples of stronger hybrid dysfunction in females. Indeed, female hybrids of *Neodiprion* sawflies show greater inviability than do hybrid males (Bendall et al., 2023; Table 1), though the sex bias is the reverse in hybrids of *Nasonia* wasps (Koevoets and Beukeboom, 2009; Koevoets et al., 2012a) with indirect evidence in several species of haplodiploid ants and mites also consistent with greater dysfunction of male hybrids (reviewed in Bendall et al., 2023). It remains to be established confidently whether exceptions to classic Haldane’s rule are any more common in haplodiploids than in taxa with heteromorphic sex chromosomes. Cytonuclear incompatibilities also present an important potential source of sex-biased hybrid dysfunction in haplodiploids, as implicated in data from *Nasonia* (Koevoets et al., 2012a), in contrast to diplodiploids in which hemizygous sex chromosomes comprise a small minority of the nuclear genome (Presgraves, 2002). Moreover, the longer-term consequences of Haldane’s rule differ for haplodiploid taxa relative to diploids, such that patterns of biased introgression of mitochondrial: nuclear DNA are unaffected by sex-biased hybrid dysfunction in haplodiploids (Patten et al., 2015). The presence of distinct sexes allows a number of other potential explanations to be tested in haplodiploid taxa (e.g. faster male, fragile male) (Figure 2). Because males produce sperm through a mitosis-like process, rather than standard meiosis, it merits further consideration as to whether or not the fragile male hypothesis ought to apply to haplodiploids. Studies to date on haplodiploid taxa suggest that hybrid sterility evolves slowly compared to hybrid inviability (Bendall et al., 2023) and it remains to be determined empirically whether any consistent trends for sex-biased hybrid sterility emerge.

### Homomorphic GSD

Approximately 5% of animals have separate sexes that develop through genetic sex determination despite not having distinguishable sex chromosomes (Bachtrog et al., 2014). An implication of such homomorphic GSD is that most of the chromosomes on which sex-determination loci reside function as an autosome. Among dioecious plants, homomorphic sex chromosomes appear to be the norm, and heteromorphic sex chromosomes are the exception (Bachtrog et al., 2014; Filatov, 2015). Consequently, dominance theory is insufficient to explain sex biases in hybrid dysfunction in such taxa because essentially the entire nuclear genome is encoded autosomally. Other models that depend on distinctive sex chromosomes also cannot explain sex-biased hybrid dysfunction in such taxa (i.e. faster X/Z; dosage compensation; gametic selection; sex-chromosome-mediated drive, translocations, incompatibilities, mis-segregation, or interaction with parental effects), unless the sex-determination loci themselves exert such pleiotropic effects. For these reasons, it has been suggested that Haldane’s rule in the generalized sense may be less universal, or may evolve at a slower pace, in frogs and other organisms that often have homomorphic GSD (Dufresnes and Crochet, 2022; Wang et al., 2023). Perhaps consistent with this idea, *Tigriopus* copepods show no evidence of disproportionate F1 hybrid sterility in males (Willett, 2008) and the genetic architecture of F2 hybrid male sterility is population-dependent (Olsen et al., 2023). Some evolutionary processes may nonetheless be important in driving sex biases in hybrid dysfunction. For example, the higher incidence of hybrid sterility in male versus female *Aedes* mosquitoes that have homogametic GSD is consistent with faster male and fragile male hypotheses (Presgraves and Orr, 1998). Similarly, toads often show sex-biased hybrid dysfunction in spite of generally having homomorphic sex chromosomes (Table 1), though possibly ZW in some species of *Bufo* (Malone and Fontenot, 2008a). Hybrid male toads frequently are inviable or sterile whereas females generally are fertile, albeit with a twofold greater incidence of female hybrid inviability (Malone and Fontenot, 2008a). Consequently, differences in the balance of countervailing factors may contribute to this variation among hybrid crosses in the sign and magnitude of sex-biased hybrid dysfunction. In principle, sexual transformation or cytoplasmic drive also could contribute to sex-biased hybrid dysfunction in taxa with homogametic GSD.

### ESD

Environmental sex determination that depends on temperature, or other cues or stressors, occurs in diverse animal phyla, most famously in reptiles and fish (Korpelainen, 1990; Valenzuela and Lance, 2004). Similar to taxa with homomorphic GSD, the entire nuclear genome is autosomal. Ancestrally in reptiles, males develop from eggs incubated at intermediate temperatures (e.g. crocodilians, some lizards, and turtles), though in most turtles males develop from cool incubation temperatures and, in ESD fish, warm incubation leads to male development (also, e.g. in tuatara) (Valenzuela and Lance, 2004; Ospina-Alvarez and Piferrer, 2008; Lawson and Rollinson, 2021). These temperature-dependent responses also are typical in species with sex determined jointly by genetic and temperature effects (Navarro-Martín et al., 2011). The genetic perturbation of a hybrid genome may disrupt the environmental response curve of sexual development to cause predictably skewed sex ratios or sex-biased defects of development.

Even species with heteromorphic sex chromosomes can show an influence of temperature, and perhaps other environmental variables, on sex-biased hybrid dysfunction (Hutter et al., 1990; Wade et al., 1999; Bundus et al., 2015). Temperature responses in vertebrate sex determination are, at least in part, often mediated by differential DNA methylation of the aromatase gene Cyp19a (Navarro-Martín et al., 2011). Those environmental conditions that are least stressful typically are predicted to favor male development, due to greater sensitivity of male fitness to variation in body condition (Trivers and Willard, 1973; Charnov and Bull, 1977; Charnov and Bull, 1989; Rowe and Houle, 1996; Lawson and Rollinson, 2021), reminiscent of the assumptions underlying the fragile male hypothesis for Haldane’s rule (see above).

As a consequence of the preceding factors, to the extent that a hybrid genome introduces a form of stress, we might predict that hybrids of taxa with ESD would show female-biased sex ratios and greater male infertility, even under otherwise benign rearing conditions. Further modeling of ESD with respect to hybrids, as well as assessment of thermal response curves in hybrids, would help clarify whether such predictions might hold for all response profiles and life histories, as short-lived species or those with males produced under warm conditions might differ in expectations (Lawson and Rollinson, 2021). For example, *Lepomis* sunfish hybrids tend to show male-biased sex ratios that are not due to the inviability of hybrid females (Bolnick, 2009; Table 1), with male-biased sex ratios also arising within sunfish species under high temperatures experienced in early development (Wang et al., 2014). Sex-biased hybrid dysfunction might also occur in ESD taxa as a result of faster male evolution in the narrow sense, due to DMIs involved in disproportionate divergence of loci with male-specific expression. Biological stress can differentially affect both sexual development (Lawson and Rollinson, 2021) and viability (Teder and Kaasik, 2023), which may also link to sexual size dimorphism (De Lisle and Rowe, 2013). Consequently, it also will be interesting to explore the interspecies hybrid context of how sexual size dimorphism and sensitivity to stress might predict Haldane’s rule and sex-biased hybrid dysfunction more generally.

### Hermaphroditism

Populations of most plants and many animals are composed of just a single-sex, hermaphrodites (Figure 1), which have fully autosomal genomes. Nonetheless, hermaphrodites can reproduce sexually, meaning that they reproduce through the fertilization of large and mostly-sedentary (female) gametes by small and motile (male) gametes that get produced either simultaneously or sequentially by a given individual over the life cycle (Ghiselin, 1969; Schärer et al., 2014; Kuwamura et al., 2020). In this way, researchers can consider sex bias in hybrid dysfunction even in hermaphroditic taxa by examining the different gametes and any distinctive reproductive structures that are associated with them (Rieseberg and Blackman, 2010). In addition to mechanisms of CMS, non-drive genetic interactions can yield hybrid male (sperm, pollen) sterility, as in rice, tomato, and *Primulina* (Long et al., 2008; Moyle and Nakazato, 2008; Feng et al., 2020). Some hermaphrodite organisms, however, do not appear to show a strong sex bias to hybrid dysfunction, such as *Solanum* tomatoes (Moyle and Nakazato, 2008). In an animal, hermaphroditic *Argopecten* scallops show sex-biased hybrid sterility primarily due to disruption of testis development and sperm fertility, though egg fertility also is compromised (Wang et al., 2017; Yu et al., 2023; Table 1). Haploid cells in hermaphrodite plant pollen are renowned for their gene expression that can manifest sex-biased effects (Rutley and Twell, 2015; Somers and Nelms, 2023), though there also is growing recognition of haploid expression in animal sperm (Joseph and Kirkpatrick, 2004; Immler, 2019; Bhutani et al., 2021). The renewed recognition and investigation of sexual selection and sexual conflict in hermaphrodites, in both plants and animals (Abbott, 2011; Lankinen and Karlsson Green, 2015; Schärer et al., 2014), highlights these distinct male and female sexual functions and their implications for speciation (Schilthuizen et al., 2011; Ågren, 2013; Cutter, 2019).

The causes of any sex-biased hybrid dysfunction in hermaphrodites must stem from mechanisms that do not depend on sex chromosomes. Consequently, faster male, fragile male, and cytoplasmic drive theories all are applicable to sex-biased hybrid dysfunction in hermaphrodites (Figure 2). It remains to be assessed broadly how common male-biased hybrid dysfunction is among hermaphrodite taxa and what relative role different theories might play as causes. Moreover, the reproductive biology of hermaphrodites might motivate the development of novel hypotheses to explain sex-biased hybrid dysfunction when it occurs. In particular, novel hypotheses might derive from further consideration of haploid expression in gametophytes and the diverse reproductive modes of hermaphrodites (e.g. protandrous versus protogynous sequential hermaphroditism, monoecy, androdioecious versus gynodioecious).

## Distinguishing among mechanisms of sex-biased hybrid dysfunction

There is a broad appreciation that Haldane’s rule emerges due to the influence of multiple factors. This appreciation highlights how important it is to deduce the contributions of those distinct factors acting to either reinforce or oppose a given direction of sex-biased hybrid dysfunction (Laurie, 1997). The challenge of deciphering reinforcing or opposing factors applies to classic Haldane’s rule, and even more so for non-heterogametic taxa to control the magnitude and sign of sex-biased hybrid dysfunction. With respect to classic Haldane’s rule, the relative input of each contributing factor is key to explaining exceptions. The balance of factors also will contribute to the nature of hybrid dysfunction in terms of which aspects of development are expected to be compromised, whether they be gametes, non-germline reproductive structures, embryonic development, or other aspects of ontogeny and life history.

### Experimental manipulation of sex determination

A variety of established research approaches can help to distinguish the relative contributions of distinct factors that may lead to sex-biased hybrid dysfunction. One of the most powerful techniques applies manipulative experimentation. For example, in ESD turtles, the application of particular hormones to eggs can be used to manipulate sex development independently of temperature (Warner et al., 2017). Similarly, the sex of *Xenopus* clawed frogs can be reversed experimentally via hormone treatment to contrast sex-reversed individuals with animals that develop from the typical sex determination defined by their heteromorphic Z and W sex chromosomes. By applying this technique to *Xenopus* hybrids (Table 1), Malone and Michalak, 2008b determined that it is physiological male-ness per se and not sex chromosome composition that is primarily responsible for sex-biased hybrid dysfunction (sterility) and that the traits of female hybrid individuals (fertility) have greater organismal robustness in the face of elevated perturbations to gene expression (Malone and Michalak, 2008c). These experiments helped explain an exception to classic Haldane’s rule, consistent with fragile male and/or faster male evolution overpowering the influence of dominance.

Experimental manipulations of sex determination and sex chromosome karyotype are valuable in other systems, as well. For example, the generation of XXY female sex chromosome compositions of *Drosophila* have proven valuable since the experiments of Sturtevant in the 1920s for exploring genetic questions of sexual asymmetry in speciation (Coyne, 1985; Barbash, 2010), and were instrumental in demonstrating how the genetic causes can differ for hybrid sterility and inviability (Orr, 1993b). In haplodiploid *Nasonia* wasps, artificial diploid males can be generated to dissect the influence of dominance and other factors (Cohen et al., 2021). Artificial tetraploid *Caenorhabditis* also have demonstrated that hybrid male sterility is sensitive to sex-chromosome ploidy (Woodruff et al., 2010). Moreover, the well-characterized sex determination pathway in *C. elegans* and related nematode species offers a number of genetic opportunities for such manipulations (Hodgkin, 2002), including creating genetic models of temperature-dependent sex determination (Janzen and Phillips, 2006). These manipulative experimental approaches are underexploited for testing the relative contributions of alternative hypotheses for sex-biased hybrid dysfunction.

### Ontogenetic analysis of hybrids

The quantitative characterization of defects in development provides another means to explore the causes and consequences of sex-biased hybrid dysfunction. For example, measuring the incidence of hybrids among different age classes in wood ants allowed Kulmuni et al., 2020 to assess selection on hybrid males. Expanding temporal assessments of reproductive isolation across the full ontogeny of hybrids – as has been conducted in toads, nematodes, and flies (Malone and Fontenot, 2008a; Bundus et al., 2015; Turissini et al., 2018) – is instructive for determining how and when the sexes can differ in sensitivity to genetic perturbation to organismal development to impact hybrid fertility, viability, and other traits (Cutter and Bundus, 2020; Cutter, 2023a).

### Analysis of later generation hybrids

Hybrid dysfunction that manifests in F2 and later generations also can provide a powerful source of insight for learning about the mechanisms of sex-biased hybrid dysfunction, despite the classic Haldane’s rule emphasis on the F1 generation. For example, multi-generation crossing experiments in *Silene* helped to demonstrate how dominance theory was sufficient to explain Haldane’s rule for sex-biased hybrid sterility but not for inviability (Demuth et al., 2014). Later-generation crosses in *Tribolium* showed how incompatibility loci linked to sex-chromosomes often indicated sexually-unbiased effects (Demuth and Wade, 2007a). Genetic maps inferred from interspecies hybrid populations can point to regions of transmission ratio distortion that may involve sex-biased effects, potentially caused by genetic drive, selfish genetic elements, or strong DMIs (Fishman et al., 2001; Woodruff et al., 2010). Over longer timescales, hybrid populations (Schumer et al., 2015; Powell et al., 2020), hybrid zones (Payseur, 2010; Shurtliff, 2013), population genomic patterns of introgression (Payseur and Rieseberg, 2016; Wolf and Ellegren, 2017), or experimental evolution with hybrids (Matute et al., 2020; Szabo and Cutter, 2023) permit evaluation of sex-linked introgression as a contributor to sex-biases in reproductive isolation (Presgraves, 2018; Fraïsse and Sachdeva, 2021).

### Parent-of-origin asymmetries in sex-biased hybrid dysfunction

Reciprocal cross-experiments often reveal parent-of-origin asymmetries in the degree of hybrid dysfunction, termed Darwin’s corollary to Haldane’s rule (Turelli and Moyle, 2007). Explanations for Darwin’s corollary remain incompletely integrated with patterns and exceptions for Haldane’s rule more broadly, though the asymmetric accumulation of substitutions between lineages is expected to restrict the potential for classic Haldane’s rule to manifest (Orr, 1993a). One promising avenue is to exploit modified versions of Fisher’s geometric model of fitness landscapes to integrate parent-of-origin asymmetries with sexual asymmetries in testable ways (Simon et al., 2018; Schneemann et al., 2022). It also would be valuable to assess more explicitly what forces could generate parent-of-origin asymmetries in sex-biased hybrid dysfunction in systems lacking heteromorphic sex chromosomes (Table 2), as well as the conditions under which factors leading to Darwin’s corollary might reinforce or oppose Haldane’s rule e.g., cytonuclear incompatibilities (Arntzen et al., 2009).

**Table 2.** Outstanding questions for Haldane’s rule and sex-biased hybrid dysfunction.

Consideration	Questions in need of addressing
Model development in exploring sex-biased hybrid dysfunction	How can models of classic Haldane’s rule be extended to provide predictions for sex-biased hybrid dysfunction in taxa lacking heteromorphic sex chromosomes?
	What new models can be developed to explain sex-biased hybrid dysfunction in taxa with homomorphic GSD, ESD, haplodiploidy, and hermaphroditism?
Relative influence of factors causing Haldane’s rule	What is the balance across sexual systems of forces that reinforce and oppose hybrid dysfunction biased toward a particular sex?
	Which mechanisms most commonly explain exceptions to classic Haldane’s rule, and how do they link to phylogeny?
Associating sex-biased hybrid dysfunction with developmental timing over life history	Under what circumstances do alternative mechanisms for sex-biased hybrid dysfunction affect predictions differently for sterility than for inviability?
	Is sex-biased hybrid dysfunction truly more prevalent for sterility than for inviability, and if so, why?
	To what extent does sex bias in pre-zygotic reproductive isolation associate with sex bias in post-zygotic hybrid dysfunction?
Connecting to other features of reproductive isolation	How do predictions for sex-biased hybrid dysfunction intersect with parent-of-origin asymmetries (Darwin’s corollary to Haldane’s rule)?
	What is the extent and biological significance of within-species genetic variation for sex-biased hybrid dysfunction?
Approaches and methodologies to investigate sex-biased hybrid dysfunction	What is the relative contribution of divergence in regulatory and coding sequence to sex-biased hybrid dysfunction, and how do they conform to alternate theoretical expectations?
	How might inference of different rates of accumulation of sex-biased hybrid dysfunction from ‘speciation clock’ analysis inform alternate explanatory models?
	How might taxa with multiple sex chromosomes, neo-sex chromosomes, and autosomal paleo-sex chromosomes be exploited to inform alternate models for sex-biased hybrid dysfunction?
	How can analysis of hybrid zones and hybrid populations differentiate among alternative explanations for sex-biased hybrid dysfunction?
	What do experimental manipulations of sex using hormone treatments, transgenic alterations, and karyotype perturbations indicate for general causes of sex-biased hybrid dysfunction?
	How can alternative causes of sex-biased hybrid dysfunction be distinguished by testing for differences in the ontogenetic timing at which dysfunctional development manifests?

### Sex-biased hybrid transcriptome misexpression

Transcriptome-wide gene expression perturbation in hybrids is now an accessible way to quantify sex-biased trait dysfunction (i.e. genes showing transgressive misexpression phenotypes) (Mack and Nachman, 2017; Cutter, 2023a; Runemark et al., 2024). Differences between the sexes in the magnitude, extent (i.e. number of loci), and kind (i.e. due to *cis*-acting and/or *trans*-acting regulatory divergence) of hybrid misexpression are readily observed in a variety of taxa (Landry et al., 2007; Malone and Michalak, 2008c; Mank, 2017; Signor and Nuzhdin, 2018; Sánchez-Ramírez et al., 2021). In principle, such information on expression and regulatory divergence may help to inform how likely is a faster X/Z, faster male, or fragile male explanation for Haldane’s rule on dysfunctional organismal traits. Even in species with homomorphic sex chromosomes with pseudo-autosomal gene content, however, expression of genes linked to the same chromosome as the sex-determining region can show pronounced sex biases (Vicoso et al., 2013), although it is not known what occurs in hybrids of such taxa. A challenge of such analyses, however, is to interpret how the degree of misexpression differences between the sexes may translate into sex differences in organismal hybrid dysfunction (Malone and Michalak, 2008c) and how misexpression itself may represent the consequence rather than the cause of hybrid defects that originated earlier in development (Ortíz-Barrientos et al., 2007; Kerwin and Sweigart, 2020).

Given the observation that expression divergence and coding sequence divergence often are decoupled (Castillo-Davis et al., 2004; Tirosh and Barkai, 2008; Sánchez-Ramírez et al., 2021; Kopania et al., 2022), it will be interesting to assess the relative importance of regulatory and coding changes as causes of Haldane’s rule. Among ‘speciation genes’ described to-date (Johnson, 2010; Presgraves, 2010b; Maheshwari and Barbash, 2011), there are examples of DMIs mediated by both regulatory and coding differences between species. A larger sample of the molecular causes, however, is required to draw general conclusions about the relative contribution of regulatory and coding divergence to alternative explanations for sex-biased hybrid dysfunction.

### Reproductive isolation clocks

**Figure 3.**
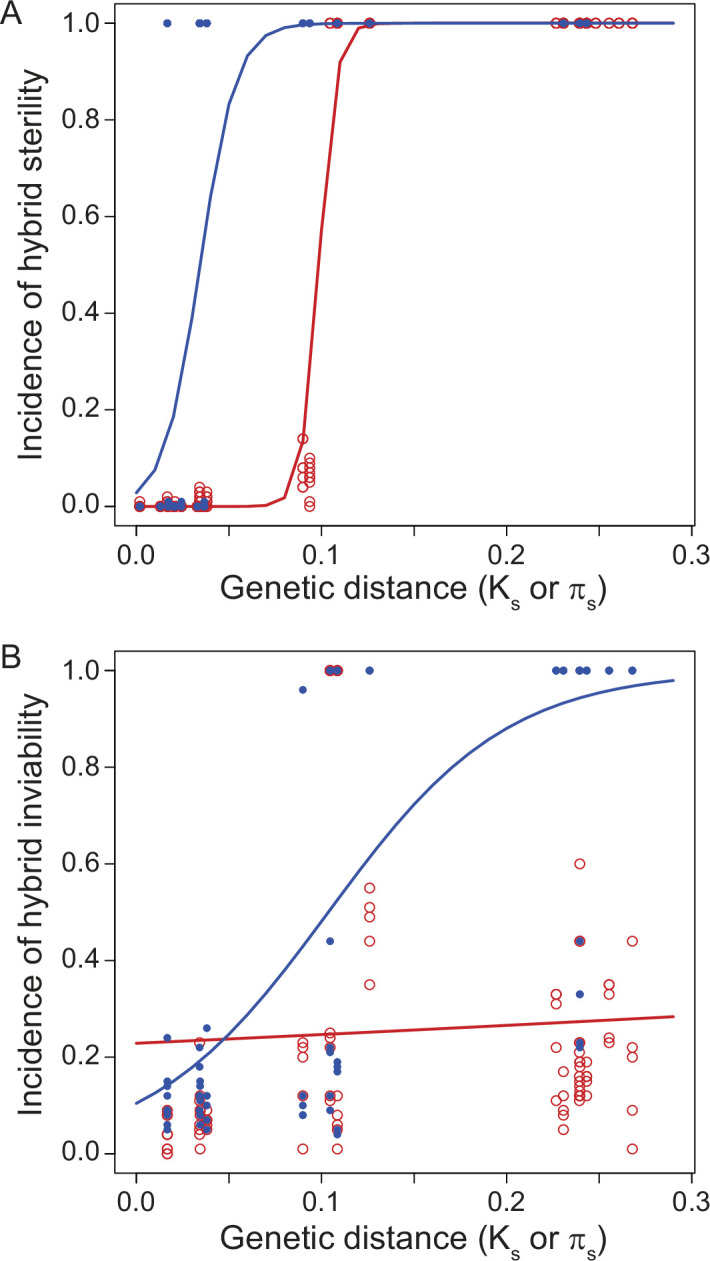
Reproductive isolation clocks for male (blue) and female (red) post-zygotic reproductive isolation documents the accumulation of sex-specific hybrid dysfunction over population divergence. Hybrid sterility (**A**) and hybrid inviability (**B**) evolve sooner for males than for females, on average, in *Drosophila*. Figure is redrawn from Turissini et al., 2018.

Another instructive way to investigate speciation is by building ‘reproductive isolation clocks’ that quantify reproductive isolation between species as a function of the duration of divergence between them Figure 3. Pioneered in the classic 1989 study of *Drosophila* by Coyne and Orr, 1989, this approach has now been applied to a variety of taxa for distinct types of reproductive isolation traits (Coyne and Orr, 1997; Sasa et al., 1998; Presgraves, 2002; Price and Bouvier, 2002; Bolnick and Near, 2005; Phillips and Edmands, 2012; Lima, 2014; Turissini et al., 2018; Coughlan and Matute, 2020; Melander and Mueller, 2020). These kinds of analyses show that classic Haldane’s rule appears especially readily between those species pairs with less divergence between them (Coyne and Orr, 1997), although Haldane’s rule also occurs between highly divergent species (Baird et al., 1992). In some taxa (e.g. flies), hybrid sterility tends to evolve sooner than hybrid inviability (Figure 3), whereas in other taxa it is the reverse (e.g. wasps) (Koevoets and Beukeboom, 2009; Presgraves, 2010a). The greater pertinence of some factors as a contributor to sex-biased hybrid dysfunction in terms of fertility versus viability (e.g. sterility via faster male and fragile male theories, inviability via maternal effects) could benefit from further integration with speciation clock analysis. As such studies grow in availability, it will be important to account for the expectation of more idiosyncratic cases at low divergence due to the stochasticity of fewer genetic incompatibilities. Even if most X-linked incompatibilities act recessively, nonetheless, some will be dominant (Laurie, 1997; Moran et al., 2017). Stochasticity in the effects of substitutions at short timescales of divergence means that any X-linked dominant incompatibilities may arise in a way that interacts with divergence time to exacerbate idiosyncrasies and exceptions to Haldane’s rule at short timescales of divergence.

Dominance theory (and faster-X/Z theory) predicts that taxa will evolve sex-biased hybrid dysfunction more quickly if they have sex chromosomes that comprise a larger portion of the total genome (Turelli and Orr, 1995; Turelli and Begun, 1997). Species with heteromorphic sex chromosomes do tend to show stronger Haldane’s rule than those with homomorphic sex chromosomes (Phillips and Edmands, 2012; Lima, 2014), and taxa with a greater proportion of their genomes being comprised of heteromorphic sex chromosomes also tend to show stronger reproductive isolation (Turelli and Begun, 1997). We generally expect stronger sex-biased hybrid dysfunction in taxa with heteromorphic sex chromosomes because the possible paths to hybrid dysfunction that uniquely depend on sex chromosomes are so multifarious (Dufresnes and Crochet, 2022). This expectation connects classic Haldane’s rule and the large-X effect, sometimes requiring careful consideration to disambiguate the large-X/Z effect from faster-X/Z evolution (Delph and Demuth, 2016; Cowell, 2023). Contrasts in rates of evolution for sex-biased effects in hybrids are especially amenable to comparative analysis within a reproductive isolation clock framework (Phillips and Edmands, 2012; Lima, 2014).

Further consideration with other sexual systems – and a broader range of organisms more generally, including those with multiple sex-chromosomes (e.g. X_1_X_2_Y_1_Y_2_ or X_1_X_2_OO), neo-sex chromosomes, or autosomal paleo-sex chromosomes – would be especially enlightening in assessing the pervasiveness of such patterns and their implications for different theories for the causes and evolution of sex-biased hybrid dysfunction. For example, stickleback fish are renowned for their rapid evolution of sex determination, with some taxa having male- or female-heteromorphic sex chromosomes and others being homomorphic (Ross et al., 2009). *Pungitius* ninespine stickleback is unusual in this group in showing male-biased hybrid sterility, as a result of spermatogenesis defects (Takahashi et al., 2005). Further interrogation of this group, including through reproductive isolation clock-type analyses, may prove valuable in discriminating the relative influence of different factors to sex-biased (or, on balance, sex-unbiased) hybrid dysfunction.

### Inspiration for new theories to explain sex-biased hybrid dysfunction

Among the virtues of expanding the study systems available to inform Haldane’s rule is the opportunity that diverse systems present to inspire new ideas for how the evolution of sex-biased hybrid dysfunction comes about. For example, species with haploid expression in gametophytes suggest an intersection of dominance theory and the fragile male hypothesis, if haploid expression is more prevalent in male gametophytes. Some plant taxa also are well-known for paternal transmission of plastid genomes (Munasinghe and Ågren, 2023), in contrast to the more familiar scenario of female transmission, and so may provide compelling substrate for tests of hypotheses that depend on parental effects and evolution of cytoplasmic factors. Taxa with alternating sexual and asexual periods of reproduction may also impose evolutionary pressures distinct from obligatorily sexual organisms that lead to predictable effects of sex-biased dysfunction (or lack thereof) in hybrids.

Even for species with heteromorphic sex chromosomes, there may be room for new or modified ideas. For example, specific population genetic conditions are required to generate faster X/Z evolution (Charlesworth et al., 1987; Orr, 1993a; Turelli and Orr, 1995; Presgraves and Meiklejohn, 2021). Some taxa might differ from the assumptions in such a way as to lead to ‘slower X/Z’ evolution to be expected to influence sex-biased hybrid dysfunction, for example, if the sex chromosomes have a lower mutation rate than autosomes, lower observed dN/dS ratios, or a rarity of genes with male-biased expression. Similarly, neo-X chromosomes (Mrnjavac et al., 2023) and adaptation from standing variation (Orr and Betancourt, 2001) also can lead to ‘slower X/Z’ evolution. Such scenarios could help explain exceptions to classic Haldane’s rule. The pace of evolution of fixed DMI alleles ought to accumulate faster for some mechanisms that can contribute to sex-biased hybrid dysfunction, such as under faster-X/Z, sex-chromosome and cytoplasmic drive, and faster-male theories. Consequently, within-species variation for Haldane’s rule might also distinguish taxa in predictable ways, with polymorphisms tending to be rarer when DMIs responsible for sex-biased hybrid dysfunction accrue rapidly (Rieseberg and Blackman, 2010; Lachance et al., 2011; Cutter, 2012).

Moreover, most explanations for Haldane’s rule depend on the Dobzhansky-Muller model of incompatibilities (Box 2; Moyle et al., 2010; Delph and Demuth, 2016). Theory about the accumulation of DMIs depends on the input of newly derived mutations (Orr, 1993a; Turelli and Orr, 1995; Orr and Turelli, 1996), and so divergence from ancestral standing variation generally is not considered to be an important source of negative effects of epistasis in hybrids that could generate Haldane’s rule. Nonetheless, negative effects of epistasis between ancestral alleles is possible (Corbett-Detig et al., 2013), and could potentially provide a basis for ‘ancestral-ancestral DMIs’ arising from standing variation within an ancestral population. Consequently, the assumptions that underpin existing theory about DMI evolution raises the question of how and whether alternative details of the genetic interactions controlling DMIs might suggest new explanations for Haldane’s rule. For example, do assumptions about single-locus incompatibility versus complex multi-locus DMIs versus network models of incompatibility predispose hybrids to sex-biased dysfunction to different degrees in different sexual systems? Other genetic complications beyond locus number and interaction structure might also serve to influence sex-bias in predictable ways. For example, the observation of tissue-dependent dominance reversal in some hybrids (de Zwaan et al., 2022) raises questions about how commonly they occur and whether they might contribute substantively to models of Haldane’s rule.

Fitness landscape models (Barton, 2001; Chevin et al., 2014; Fraïsse et al., 2016; Simon et al., 2018; Schneemann et al., 2022), among other approaches (Schiffman and Ralph, 2022), have provided tractable and insightful inroads to understand expectations about hybrid fitness for a diverse range of scenarios to complement DMI models (Orr, 1993a; Turelli and Orr, 1995; Orr and Turelli, 1996). In particular, they empower predictions about sex asymmetries, cross asymmetries, and fitness dynamics as a function of accumulated divergence to permit comparisons among models with different assumptions (Schneemann and Welch, 2024). For example, perhaps non-intuitively, exceptions to Haldane’s rule may be especially likely when the X-chromosome is very large (Schneemann et al., 2022), a prediction that may be particularly relevant to situations of haplodiploidy. Such approaches also may prove valuable in bridging timescales, starting from sex differences in heterosis (hybrid vigor) at ‘optimal outbreeding’ genetic distances – with male-biased heterosis observed in organisms like beetles and mice whereas ‘homogametic heterosis’ is found in some other cases (Stonaker, 1963; Boylan and Wong, 1965; White et al., 1970). Patterns at these shorter genetic distances may then link in predictable ways to sex-biases in hybrid dysfunction and ‘outbreeding depression’ at greater genetic distances (Bolnick and Near, 2005). I anticipate that theoreticians and organismal biologists will explore genetic factors like these to devise novel and testable hypotheses, and strategies for experimental design and analysis, to distinguish the relative importance of distinct contributors to sex-biased hybrid dysfunction in taxa with shared circumstances.

Among the key outstanding questions is, what is the balance of reinforcing and opposing forces that can influence sex-biased hybrid dysfunction across sexual systems? Exceptions to classic Haldane’s rule, and their dissection (Malone and Michalak, 2008b), certainly point to this tension. But further research is required to assess the predispositions of different kinds of taxa and sexual systems to the various forces. We also seek to understand general principles across forces and taxa. For example, if Haldane’s rule is weak in most taxa that lack heteromorphic sex chromosomes, as has been proposed (Dufresnes and Crochet, 2022), then it would lend support to the notion that, in absolute terms, factors like faster male and fragile male evolution are relatively weak influences on hybrid dysfunction. Studies on taxa with heteromorphic sex chromosomes suggest this possibility (Schilthuizen et al., 2011), though a thorough evaluation of partial contributions remains to be seen. Characterization of general patterns may help to predict and explain differences in the speed of accumulation of hybrid sterility vs inviability in different taxa, e.g. Lepidoptera vs *Drosophila* (Presgraves, 2002) and amphibians vs mammals (Dufresnes and Crochet, 2022), or differences among taxa in whether they gradually accumulate DMIs or rapidly evolve RI e.g., via cytoplasmic drive mechanisms (Presgraves, 2002). Examination of sex-biased hybrid dysfunction broadly also provides more opportunities for integrating observations of sex-bias (or lack thereof) in pre-zygotic isolation and reinforcement theory. Key to assessing relative contributions of the multiple possible drivers of sex-biased hybrid dysfunction, and to going beyond the pattern of classic Haldane’s rule, involves the regularized reporting of sex differences, or lack thereof, in metrics of reproductive isolation for speciation studies for all sexual systems. Efforts to generate broad taxonomic databases of sexual systems, like the Tree of Sex initiative (Ashman et al., 2014; Bachtrog et al., 2014), combined with establishing similar resources for speciation and reproductive isolation (Stankowski et al., 2024) will provide a powerful substrate for meta-analysis to assess general features in the evolution of sex-biased hybrid dysfunction across sexual systems (Table 3).

**Table 3.** Recommended research directions to establish a generalized view of Haldane’s rule and sex-biased hybrid dysfunction.

Recommendation	Status, prospect, or approach
Compile sexual system information across taxa	Tree of Sex database in progress (Ashman et al., 2014; Bachtrog et al., 2014)
Compile sex-biased reproductive isolation information across taxa	Speciation database proposed (Stankowski et al., 2024)
Integrate speciation modeling predictions for sex bias explicitly with distinct sexual systems	Feasible for DMI, fitness landscape, systems theory, and other paradigms (Orr, 1993a; Orr and Turelli, 1996; Simon et al., 2018; Schiffman and Ralph, 2022)
Test for presence/absence of distinct sources of sex-biased hybrid dysfunction across taxa	Well-studied in Diptera and Lepidoptera, but requires further empirical study and integration more broadly
Quantify the relative contribution of distinct sources of sex-biased hybrid dysfunction	Most feasible in genetic model organisms, but diverse experimental (e.g. backcross analysis, hormone treatment) and genomic (e.g. transcriptomes, molecular evolution) techniques empower study in many taxa
Conduct developmental analyses of genetic complexity for hybrid sterility and inviability for each sex	Experimentally feasible with interspecies QTL mapping, hybrid allele-specific expression analysis, or other approaches
Characterize ‘speciation clocks’ separately for each sex in different sets of taxa	Available for some *Drosophila* (Turissini et al., 2018), feasible to test in any focal group with partial reproductive isolation between many species pairs

### Conclusions

Organisms with separate sexes and heteromorphic sex chromosomes remain important study systems for exploring speciation, and yet many principles that underlie the evolution of reproductive isolation extend more broadly across the tree of life. Consequently, I propose a broad and inclusive view of the processes that generate sex biases in hybrid dysfunction (Box 1), irrespective of sexual system, and despite the continuing importance of deciphering the mechanisms responsible for classic Haldane’s rule in the strict sense. This broader view of sex-biased hybrid dysfunction calls attention to a variety of outstanding biological questions in need of resolution from a range of concrete research directions (Table 3).
